# Corticosteroid Injection Practices of Australian Podiatrists for Common Foot and Ankle Conditions

**DOI:** 10.1002/jfa2.70214

**Published:** 2026-09-11

**Authors:** Christopher B. Couesnon, Matthew Cotchett, Naomi Blood, Glen A. Whittaker

**Affiliations:** ^1^ Discipline of Podiatry School of Allied Health, Human Services and Sport La Trobe University Melbourne Victoria Australia; ^2^ Northern Hospital Epping Victoria Australia; ^3^ Toorak Village Podiatry Toorak Victoria Australia

**Keywords:** adrenal cortex hormones, corticosteroid injection, foot joints, injections, morton neuroma, plantar fasciitis, podiatry

## Abstract

**Introduction:**

Corticosteroid injections are commonly used to manage foot and ankle conditions; however, there is limited evidence guiding corticosteroid selection, dosing, imaging guidance and injection techniques. This study aimed to describe current corticosteroid injection practices among Australian podiatrists and podiatric surgeons.

**Methods:**

A descriptive cross‐sectional survey was distributed via REDCap to Australian podiatrists and podiatric surgeons endorsed to prescribe scheduled medicines. The questions were developed using an iterative process and focused on demographic and professional characteristics, administrative factors and injection practices for plantar fascia, intermetatarsal and intra‐articular injections. The survey was conducted between January 2025 and April 2026 and reported according to the Consensus‐Based Checklist for Reporting of Survey Studies (CROSS). Descriptive statistics were used to summarise injection practices for plantar fascia, intermetatarsal and intra‐articular injections.

**Results:**

Forty‐five people participated in the study, and 33 completed the survey in full. Participants had a mean of 17.1 years in practice and 9.6 years administering corticosteroid injections. Most participants were endorsed podiatrists (64%), with the remainder being podiatric surgeons (33%). Participants reported administering a mean maximum of three corticosteroid injections per patient annually and recommending a mean interval of 3 months between injections. Betamethasone 5.7 mg/mL was the most common corticosteroid across all injection sites and was frequently combined with ropivacaine or bupivacaine. Ultrasound guidance was used by 50% of participants for plantar fascia injections, 39% for intermetatarsal injections and 46% for intra‐articular injections. Symptom relief was perceived to last between 3 and 6 months, whereas adverse effects were recalled to be infrequent and most commonly included steroid flare and post‐injection pain.

**Conclusion:**

Australian podiatrists and podiatric surgeons reported consistency in some corticosteroid injection practices and variability in others. These findings provide an initial descriptive benchmark of corticosteroid injection practice in Australian podiatry and may inform future clinical guidelines and research.

## 1 Introduction

Corticosteroid injection is commonly used in the management of musculoskeletal foot and ankle conditions [1, 2, 3] and is safe and effective in reducing inflammation, alleviating symptoms and improving function for common foot and ankle conditions such as plantar heel pain, interdigital bursa/neuroma and osteoarthritis [2, 4, 5, 6, 7, 8]. When conservative interventions fail or when surgery is contraindicated, corticosteroid injection may delay or reduce the need for surgical intervention, potentially improving patient satisfaction and reducing healthcare costs [2, 9].

In Australia, podiatrists who are endorsed to prescribe scheduled medicines can administer corticosteroid injection for foot and ankle conditions within their scope of practice. Given the substantial burden of foot and ankle pathology in the community, podiatrists are well positioned to play a central role in delivering timely corticosteroid injection therapy. Their involvement is likely to become increasingly important as demand for nonsurgical management grows [9].

Despite this, there is no evidence about how Australian podiatrists currently use corticosteroid injection in clinical practice or how these practices compare with those reported in other regions. Past research from other countries has found variability in corticosteroid injection practices across professions and clinical settings. For example, Backhouse et al. [3] examined intra‐articular use of corticosteroid injection for symptomatic first metatarsophalangeal joint osteoarthritis in the United Kingdom, whereas research by Blankstein et al. [10] explored common practices in intra‐articular corticosteroid injection for the treatment of knee osteoarthritis within the American Association of Hip and Knee Surgeons. Both studies revealed substantial variability across and within allied health and medical professions [3, 10], including variability in corticosteroid and anaesthetic selection, dosing, equipment used and use of imaging guidance [3, 10].

Understanding how Australian podiatrists use corticosteroid injection in their clinical practice can inform the design of randomised trials, help podiatrists benchmark their practice and inform guideline development. Therefore, the aim of this study is to describe corticosteroid injection practices among Australian podiatrists for common foot and ankle conditions, including administration (e.g., cost), corticosteroid and local anaesthetic medication selection, dosages and injection techniques.

## 2 Materials and Methods

A descriptive, cross‐sectional survey design was used to investigate corticosteroid injection practices among Australian podiatrists and podiatric surgeons. The study was approved by the La Trobe University Human Research Ethics Committee (HEC24151). All participants provided informed consent prior to participation. The study is reported in accordance with the Consensus‐Based Checklist for Reporting of Survey Studies [11] (Supporting Information S1).

### 2.1 Participants

Australian podiatrists and podiatric surgeons with an endorsement to prescribe scheduled medicines were eligible to participate in the online survey. Participants were required to be (i) a podiatrist or podiatric surgeon with an endorsement to prescribe scheduled medicines registered with the Australian Health Practitioner Regulation Agency, (ii) an Australian resident, (iii) able to understand written English and (iv) administer at least one corticosteroid injection in the foot and ankle within the past 6 months.

A convenience sampling approach was used to recruit participants. According to the most recent data from the Podiatry Board of Australia, there are approximately 275 podiatrists with an endorsement to prescribe scheduled medicines in Australia [12]. However, the total number of podiatrists and podiatric surgeons regularly administering injections in Australia is unknown; therefore, a formal sample size calculation was not feasible. Participants were recruited via email invitations, social media posts through La Trobe University Podiatry platforms and distribution to members of the Australian Podiatry Association. Individuals who completed the survey were also encouraged to share the survey link with colleagues who may be interested in participating. Participation was voluntary and anonymous.

### 2.2 Survey Design

The survey was originally designed to collect responses from several health professionals but was subsequently revised to focus on podiatrists and podiatric surgeons. Survey questions were developed through an iterative process involving a literature review, initial question development by two authors (GW and NB) and refinement of the survey questions through collaboration with relevant podiatrists and podiatric surgeons with an endorsement to prescribe scheduled medicines who regularly administer corticosteroid injections in the foot and ankle. The refinement included feedback on content, clarity and usability, which helped refine the final version (Supporting Information S2). The survey collected data across three categories: (i) demographic and professional characteristics (e.g., age, years in practice and training background), (ii) administrative factors (e.g., injection frequency, recommended intervals and cost) and (iii) clinical practices for plantar fascia, intermetatarsal and intra‐articular injections (e.g., corticosteroid and local anaesthetic medication selection, dosing, injection technique, use of imaging guidance, duration of effect and adverse events). Plantar fascia, intermetatarsal and intra‐articular injections were chosen because they were considered by the research team to be the most commonly performed corticosteroid injections by Australian podiatrists, encompass the most common musculoskeletal foot pathologies encountered in practice and allow the survey to remain concise and maximise completion rates.

### 2.3 Data Analysis

Data were collected using REDCap (Research Electronic Data Capture), a secure web‐based platform hosted at La Trobe University [13]. The survey link permitted one response per participant. Data were analysed descriptively using Microsoft Excel. To maximise data use, partially completed surveys were retained. Continuous data were summarised as means and standard deviations (SD), whereas medians and interquartile ranges were used for non‐normally distributed data. Categorical variables were reported as totals and percentages. Free‐text responses were analysed using a descriptive content analysis approach, whereby responses were reviewed and the most frequently occurring themes or categories were reported. The average cost of injection was calculated as the mean of the total out‐of‐pocket cost to the patient that each participant reported charging.

## 3 Results

The survey was accessible from 6 January 2025, and data collection ceased when we had exhausted responses through our recruitment methods. Data collection was closed on 30 April 2026. The total number of online survey links distributed was unknown; however, 66 individuals accessed the survey. Of these, 45 consented to participate, and 33 (73%) completed the survey in full. Most participants were podiatrists with an endorsement to prescribe scheduled medicines (64%), whereas the remainder were podiatric surgeons (33%).

Training pathways for corticosteroid administration were diverse, with participants frequently reporting multiple forms of education. Mentoring (76%) and peer support (60%) were the most commonly reported learning methods, followed by self‐directed learning (42%), award courses (40%) and nonaward courses (40%). Participants reported substantial clinical experience, with a mean duration of practice of 17.3 years and a mean of 9.6 years administering corticosteroid injections. Demographic characteristics are presented in Table 1.

**TABLE 1:** Participant characteristics.

	Mean or total (% or SD) *n* = 45
Age, years	41.4 (9.5)
Gender	
Male	31 (70%)
Female	12 (27%)
Prefer not to answer	2 (5%)
State/territory of practice	
Victoria	20 (44%)
New South Wales	12 (27%)
Western Australia	7 (16%)
South Australia	3 (7%)
Queensland	2 (4%)
Australian Capital Territory	1 (2%)
Northern Territory	0
Culture/ethnicity	
Australian	16 (42%)
British	5 (13%)
Lebanese	4 (11%)
Irish	3 (8%)
Italian	3 (8%)
Turkish	2 (5%)
Greek	1 (3%)
Egyptian	1 (3%)
Iranian	1 (3%)
Vietnamese	1 (3%)
South African	1 (3%)
Dutch	1 (3%)
Occupation	
Podiatrist with an endorsement to prescribe scheduled medicines	29 (64%)
Podiatric surgeon	15 (33%)
Training pathways	
Mentoring	34 (76%)
Peer support	27 (60%)
Self‐taught	19 (42%)
Award course	18 (40%)
Nonaward course	18 (40%)
Years in podiatry practice	17.3 (10.2)
Years administering corticosteroid injection	9.6 (8.7)

Abbreviation: SD = standard deviation.

Clinical and administrative considerations are presented in Table 2. Participants reported a mean maximum of three corticosteroid injections per year for a single patient, with a recommended mean interval of 3 months between injections. The mean patient out‐of‐pocket cost was AUD 188.18. Pain was the most influential factor guiding corticosteroid injection decision‐making (50%).

**TABLE 2:** Administrative and clinical considerations.

	Mean or total (% or SD; range) *n* = 45
Maximum corticosteroid injections for a single patient per year, years	3.0 (1.24; 2–10)
Recommended interval between injections, months	3.0 (1.77 1–12)
Patient out‐of‐pocket costs (AUD)^a^	$188.18 ($61.44; $80‐$350)
Patient factors guiding injection appropriateness	
Pain	23 (50%)
Supporting evidence	9 (20%)
Medical history	8 (18%)
Patient preference	6 (13%)
Occupation	1 (3%)
Medication history	1 (3%)
Outcome	1 (3%)
Assistant present during the injection	
Yes	6 (15%)
No	39 (98%)

Abbreviations: AUD = Australian dollars; SD = standard deviation.

^a^ Costs were calculated as the mean out‐of‐pocket cost charged by participants when administering an injection.

### 3.1 Plantar Fascia Injection

A summary of plantar fascia corticosteroid injection practices is presented in Table 3. Betamethasone 5.7 mg/mL was the most commonly selected corticosteroid (53%), with most participants administering a 1‐mL corticosteroid volume (88%) and combining the injection with a local anaesthetic (97%). Ropivacaine (32%) and bupivacaine (32%) were the most frequently selected local anaesthetics, with 1‐ and 2‐mL volumes most commonly reported.

**TABLE 3:** Practice for plantar fascia corticosteroid injection.

	Mean or total (% or SD; range) *n* = 34
Administer plantar fascia injection	
Yes	34 (76%)
No	0
Did not answer	11 (24%)
Plantar fascia injections administered per month	
0–5	27 (79%)
6–10	6 (18%)
≥ 15	1 (3%)
Corticosteroid selected	
Betamethasone 5.7 mg/mL	18 (53%)
Methylprednisolone acetate 40 mg/mL	5 (15%)
Triamcinolone acetonide 40 mg/mL	4 (12%)
Triamcinolone acetonide 10 mg/mL	4 (12%)
Dexamethasone phosphate 4 mg/mL	3 (9%)
Corticosteroid volume	
0.5 mL	2 (6%)
1 mL	30 (88%)
1.5 mL	1 (3%)
2 mL	1 (3%)
≥ 2.5 mL	0
Mix corticosteroid and local anaesthetic	
Yes	33 (97%)
No	1 (3%)
Local anaesthetic selected	
Ropivacaine	11 (32%)
Bupivacaine	11 (32%)
Lidocaine 2%	7 (21%)
Lidocaine 1%	4 (12%)
Local anaesthetic volume	
0.5 mL	1 (3%)
1 mL	11 (32%)
1.5 mL	3 (9%)
2 mL	10 (29%)
≥ 2.5 mL	8 (24%)
Did not answer	1 (3%)
Needle approach	
Medial heel	15 (44%)
Medial oblique	9 (26%)
Plantar heel	9 (26%)
Other	1 (3%)
Regional nerve block	
Yes	21 (62%)
No	13 (38%)
Topical anaesthetic	
Yes	2 (6%)
No	32 (94%)
Diagnostic imaging prior to injection	
Yes	25 (74%)
No	9 (26%)
Type of diagnostic imaging prior to injection	
Ultrasound	23 (68%)
MRI	1 (3%)
Fluoroscopy	1 (3%)
Did not answer	9 (27%)
Ultrasound guidance during injection	
Yes	17 (50%)
No	17 (50%)
Perceived duration of pain reduction	
1 month	3 (9%)
2 months	6 (18%)
3 months	9 (26%)
6 months	9 (26%)
12 months	3 (9%)
24 months	4 (12%)
Perceived frequency of adverse effects	7.2% (5.7%; 0%–20%)

Abbreviation: SD = standard deviation.

The medial heel approach was the most commonly reported injection technique (44%). Regional nerve blocks (i.e., a tibial block) were used by 62% of participants, whereas topical anaesthesia prior to injection was infrequently used (6%). Diagnostic imaging was frequently incorporated (74%), predominantly ultrasound (68%), whereas half (50%) of participants reported using ultrasound guidance during injection.

Most participants reported pain reduction lasting between 3 (26%) and 6 months (26%). The mean reported frequency of adverse effects was 7.2%, with free‐text responses indicating that the most common adverse event was a steroid flare.

### 3.2 Intermetatarsal Injection

A summary of intermetatarsal corticosteroid injection practices is presented in Table 4. Betamethasone was the most commonly selected corticosteroid (58%), with most participants administering a 1‐mL corticosteroid volume (90%) in combination with a local anaesthetic (94%). Bupivacaine was the most frequently selected local anaesthetic (35%), with 1‐mL volumes most commonly reported (55%).

**TABLE 4:** Practice for intermetatarsal corticosteroid injection.

	Mean or total (% or SD; range) *n* = 34
Administer intermetatarsal injection	
Yes	31 (70%)
No	3 (7%)
Did not answer	11 (25%)
Intermetatarsal injections administered per month	
0–5	22 (71%)
6–10	5 (16%)
11–15	3 (10%)
≥ 15	1 (3%)
Corticosteroid selected	
Betamethasone 5.7 mg/mL	18 (58%)
Triamcinolone acetonide 10 mg/mL	6 (19%)
Methylprednisolone acetate 40 mg/mL	3 (10%)
Dexamethasone phosphate 4 mg/mL	3 (10%)
Triamcinolone acetonide 40 mg/mL	1 (3%)
Corticosteroid volume	
0.5 mL	3 (10%)
1 mL	28 (90%)
1.5 mL	0
2 mL	0
≥ 2.5 mL	0
Mix corticosteroid and local anaesthetic	
Yes	29 (94%)
No	2 (6%)
Local anaesthetic selected	
Bupivacaine	11 (35%)
Ropivacaine	9 (29%)
Lidocaine 2%	5 (16%)
Lidocaine 1%	4 (13%)
Did not answer	2 (6%)
Local anaesthetic volume	
0.5 mL	1 (3%)
1 mL	17 (55%)
1.5 mL	4 (13%)
2 mL	2 (6%)
≥ 2.5 mL	5 (16%)
Did not answer	2 (6%)
Needle approach	
Dorsal	29 (94%)
Webspace	2 (6%)
Regional nerve block	
No	31 (100%)
Topical anaesthetic	
No	30 (97%)
Yes	1 (3%)
Diagnostic imaging prior to injection	
Yes	20 (65%)
No	11 (35%)
Type of imaging prior to injection	
Ultrasound	19 (61%)
MRI	1 (3%)
Did not answer	11 (35%)
Ultrasound guidance during injection	
Yes	12 (39%)
No	19 (61%)
Perceived duration of pain reduction	
1 month	3 (10%)
2 months	5 (16%)
3 months	11 (35%)
6 months	9 (29%)
12 months	2 (6%)
24 months	1 (3%)
Perceived frequency of adverse effects	6.9% (7.2%; 0%–30%)

Abbreviation: SD = standard deviation.

A dorsal approach was the predominant injection technique (94%). No participants reported using regional nerve blocks, whereas topical anaesthesia prior to injection was infrequently used (3%). Diagnostic imaging was used by 65% of participants, most commonly ultrasound (61%) and most relied on landmark‐guided injections (61%).

Most participants reported pain reduction lasting between 3 (35%) and 6 months (29%). The mean reported frequency of adverse effects was 6.9%, with free‐text responses indicating that the most common adverse events were steroid flares and post‐injection pain.

### 3.3 Intra‐Articular Injection

A summary of intra‐articular corticosteroid injection practices is presented in Table 5. Betamethasone 5.7 mg/mL was the most commonly selected corticosteroid (43%), with most participants administering a 1‐mL corticosteroid volume (71%). Almost all participants (93%) combined corticosteroids with a local anaesthetic, most commonly ropivacaine (29%) or bupivacaine (29%). The most commonly reported local anaesthetic volume was 0.5 mL (32%).

**TABLE 5:** Practice for intra‐articular corticosteroid injection.

	Mean or total (% or SD; range) *n* = 33
Administers intra‐articular injection	
Yes	28 (62%)
No	5 (11%)
Did not answer	12 (27%)
Intra‐articular injections administered per month	
0–5	22 (79%)
6–10	4 (14%)
11–15	1 (4%)
≥ 15	1 (4%)
Corticosteroid selected	
Betamethasone 5.7 mg/mL	12 (43%)
Methylprednisolone acetate 40 mg/mL	5 (18%)
Triamcinolone acetonide 40 mg/mL	6 (21%)
Triamcinolone acetonide 10 mg/mL	4 (14%)
Dexamethasone phosphate 4 mg/mL	1 (4%)
Corticosteroid choice is altered by joint	
Yes	8 (29%)
No	20 (71%)
Corticosteroid volume	
0.5 mL	7 (25%)
1 mL	20 (71%)
1.5 mL	0
2.0 mL	0
≥ 2.5 mL	1 (4%)
Mix corticosteroid and local anaesthetic	
Yes	26 (93%)
No	2 (7%)
Local anaesthetic selected	
Ropivacaine	8 (29%)
Bupivacaine	8 (29%)
Lidocaine 2%	6 (21%)
Lidocaine 1%	4 (14%)
Did not answer	2 (7%)
Local anaesthetic volume	
0.5 mL	9 (32%)
1 mL	8 (29%)
1.5 mL	1 (4%)
2 mL	4 (14%)
≥ 2.5 mL	4 (14%)
Did not answer	2 (7%)
Regional nerve block	
No	28 (100%)
Topical anaesthetic	
Yes	1 (4%)
No	27 (96%)
Diagnostic imaging prior to injection	
Yes	22 (79%)
No	6 (21%)
Type of imaging prior to injection	
Ultrasound	12 (43%)
X‐ray	4 (14%)
MRI	3 (11%)
Fluoroscopy	1 (4%)
Other	2 (7%)
Did not answer	6 (21%)
Ultrasound guidance during injection	
Yes	13 (46%)
No	15 (54%)
Perceived duration of pain reduction	
1 months	4 (14%)
2 months	3 (11%)
3 months	6 (21%)
6 months	11 (39%)
12 months	3 (11%)
24 months	1 (4%)
Perceived adverse effects frequency	4.9% (SD 3.9%; 0%–15%)

Abbreviation: SD = standard deviation.

No participants reported using regional nerve blocks, and 4% of participants reported using topical anaesthetics prior to injection. Diagnostic imaging was frequently incorporated (79%), with ultrasound being the most commonly reported modality (43%). Just over half of the participants opted for landmark‐guided injections (54%).

Free‐text responses indicated varied injection techniques; however, joint capsule infiltration was the most commonly described technique. Most participants reported pain reduction lasting approximately 6 months (39%). The mean reported frequency of adverse effects was 4.9%, with free‐text responses indicating that the most common adverse event was steroid flare and post‐injection pain.

## 4 Discussion

This study provides the first description of corticosteroid injection practices among Australian podiatrists and podiatric surgeons for common foot and ankle conditions. Participants demonstrated variability in training pathways, imaging use, injection approaches and medication selection. However, several consistent practice patterns were identified. Across all injection sites, betamethasone was the most commonly selected corticosteroid, frequently combined with ropivacaine or bupivacaine. Most participants perceived the therapeutic benefit lasting between 3 and 6 months, whereas adverse events were infrequent and most commonly consisted of steroid flare or post‐injection pain.

Our study provides the first description of the maximum number of corticosteroid injections per patient per year in the foot and ankle, with participants reporting that they administer a mean maximum of 3. These practices align with the recommendations from the Australian Therapeutic Guidelines, which suggest a maximum of four injections per joint per year to reduce associated risks [8]. None of the prior foot and ankle or multidisciplinary studies explored comparable data, as they examined the total volume of injections per year of the clinicians rather than patient‐ or joint‐level limits [3, 14, 15].

The mean interval between corticosteroid injections among our participants was 3 months, mirroring findings from Blankstein et al.’s survey of the American Association of Hip and Knee Surgeons, where most respondents recommended a minimum 3‐month interval between injections for knee osteoarthritis [11]. These findings are broadly consistent with the mean maximum number of injections per patient and injection intervals reported in our cohort.

To our knowledge, this study is also the first to quantify patient out‐of‐pocket costs for corticosteroid injections in Australian podiatric practice, with a mean reported cost of AUD 188.18 per injection. A further novel contribution of this study is the exploration of patient‐centred considerations and factors influencing the administration of a corticosteroid injection. Pain severity was the primary factor influencing corticosteroid injection decision‐making, followed by supporting evidence [16]. These findings suggest that symptom severity remains a key determinant for corticosteroid administration in clinical practice.

Betamethasone was the most commonly selected corticosteroid across all injection sites, contrasting with previous international studies where methylprednisolone and triamcinolone were more commonly used, particularly for intra‐articular injection [3, 10, 14, 15]. Differences in corticosteroid selection may reflect regional prescribing practices, medication availability, clinician training or cost considerations [15]. Despite international variability in corticosteroid selection, corticosteroid volume was relatively consistent in our cohort, with 1 mL most commonly administered regardless of injection site, consistent with previous international survey data [3, 15].

Local anaesthetics were frequently combined with corticosteroids across all injection sites, consistent with recommendations from the European Alliance of Associations for Rheumatology and previous international studies in the foot and ankle and knee [3, 10, 14, 15, 17]. Ropivacaine and bupivacaine were the most commonly selected local anaesthetics in our cohort, whereas lidocaine was more frequently reported in previous international studies [3, 14]. Levobupivacaine was commonly reported in Backhouse et al. [3] but was not used by participants in our cohort. These differences may be attributable to factors such as medication availability, access, costs and differences in prescribing practices within Australia. The use of topical anaesthesia and nerve blocks prior to corticosteroid injection varied across injection sites. More than half of the participants reported performing regional nerve blocks for plantar fascia injections, whereas none reported using them for intermetatarsal or intra‐articular injection. Only two of the participants utilised topical anaesthesia prior to injection. In contrast, Blankstein et al. [10] reported greater use of topical anaesthesia prior to injection among orthopaedic surgeons managing knee osteoarthritis.

Diagnostic imaging prior to injection was frequently used across all injection sites, being the highest for intra‐articular injection. Ultrasound was the most commonly reported imaging modality across the foot and ankle. These findings are broadly consistent with previous literature demonstrating widespread use of ultrasound for foot and ankle pathology assessment and injection planning [18, 19, 20, 21, 22]. However, unlike some previous international studies where X‐ray was more commonly utilised for intra‐articular pathology assessment, ultrasound was more frequently reported among our participants [20, 21]. Use of ultrasound guidance during injection demonstrated mixed utilisation across injection sites. Half of the participants reported using ultrasound guidance for plantar fascia and just under half for intra‐articular injections, whereas landmark‐guided injections remained more common for intermetatarsal injections. These findings are consistent with previous literature reporting a mixed use of ultrasound guidance in musculoskeletal corticosteroid injection practice [3]. This mixed utilisation likely reflects the mixed evidence on the clinical benefits of ultrasound‐guided corticosteroid injections in the foot and ankle compared with landmark‐guided injections. Past research suggests improved injection accuracy and, in some cases, superior clinical outcomes with ultrasound guidance [23, 24, 25, 26, 27, 28, 29, 30]. However, other studies report comparable pain and functional outcomes between ultrasound‐guided and landmark‐guided injections [28, 31, 32].

This is the first study to report injection approaches in the foot and ankle among Australian podiatrists and podiatric surgeons. Medial heel approaches were most frequently used for plantar fascia injection, whereas dorsal approaches predominated for intermetatarsal injection. For intra‐articular injection, many participants had not described a specific injection approach. Rather, most participants focused on aseptic sterile techniques and ensuring direct capsule infiltration. To our knowledge, there has been no research comparing different injection approaches in the foot and ankle or their impact on accuracy and clinical outcomes. Consequently, this absence suggests that clinicians are likely relying primarily on their training background, preferences or personal experience when selecting injection approaches.

Participants perceived the therapeutic benefit of injection to last between 3 and 6 months, which is consistent with previous research examining intra‐articular corticosteroid injection for knee osteoarthritis [10]. Adverse events were perceived to be infrequent by participants, with estimated rates ranging from 5% to 7% across injection sites. The most common adverse events recalled by participants were steroid flare, post‐injection pain and injection‐site discomfort, which is consistent with previous knee, foot and ankle literature [10, 14]. Collectively, these findings suggest that clinicians perceive corticosteroid injections as providing meaningful short‐ to medium‐term symptom relief with relatively low rates of adverse events.

There are some limitations to our research. First, the cross‐sectional descriptive survey design limits our ability to establish causal relationships with injection practices or observe how these practices evolve over time. Second, the absence of national data on the number of podiatrists with endorsement to prescribe scheduled medicines who are administering corticosteroid injections prevented an accurate sample size calculation. Third, the use of convenience sampling, although pragmatic for recruitment given the inability to calculate a sample size, may introduce bias. Males were overrepresented in our sample compared with the overall podiatry workforce, and this may reflect the demographic profile of clinicians working in musculoskeletal practice and podiatric surgery. Finally, our recruitment method may not have reached potential participants, which may have reduced our response rates and diversity, particularly as most respondents were only from two states in Australia. Taken together, these limitations have resulted in a potentially modest sample size, and the results may not be generalisable to the population of podiatrists and podiatric surgeons administering corticosteroid injections in Australia; however, we cannot be certain of this given the unknown population.

This study has highlighted that there is variability in corticosteroid injection practices among Australian podiatrists and podiatric surgeons, potentially due to an absence of evidence‐based guidelines. Therefore, future research should focus on randomised controlled trials to compare the clinical efficacy, safety and duration of effect of different corticosteroid and local anaesthetic formulations, injection volumes and guidance techniques. Furthermore, longitudinal studies such as repeated surveys or registry‐based data collection could track changes in corticosteroid injection practices and identify factors that influence clinicians' decisions over time. Qualitative research exploring clinician decision‐making and identifying barriers to care would provide valuable context for clinical application. Importantly, these collective suggestions for research approaches would be valuable in providing robust evidence to inform Australian best‐practice guidelines.

## 5 Conclusion

Australian podiatrists and podiatric surgeons in this sample reported broadly similar corticosteroid injection practices for common foot and ankle conditions, particularly regarding corticosteroid selection, use of local anaesthetics, injection frequency and reported duration of symptom relief. However, variation existed in training pathways, imaging use, injection techniques and patient costs, reflecting the limited availability of evidence‐based guidance specific to foot and ankle corticosteroid injections. Adverse effects were perceived to be infrequent and were recalled as most commonly steroid flare or post‐injection pain. These findings provide an initial descriptive benchmark of corticosteroid injection practice in Australian podiatry and highlight the need for further research to inform evidence‐based clinical guidelines and support consistent, high‐quality patient care.

## Author Contributions

**Christopher B. Couesnon:** formal analysis, writing – original draft, writing – review and editing. **Matthew Cotchett:** conceptualization, methodology, investigation, writing – review and editing. **Naomi Blood:** conceptualization, writing – review and editing. **Glen A. Whittaker:** conceptualization, methodology, investigation, data curation, supervision, writing – review and editing.

## Funding

This research was completed without research funding.

## Ethics Statement

Ethics approval was granted by the La Trobe University Human Research Ethics Committee (HREC) under approval number HEC24151.

## Consent

Participants were registered health professionals who provided informed consent electronically prior to commencing the online survey.

## Conflicts of Interest

The authors declare no conflicts of interest.

## Supporting information


Supporting Information S1

Supporting Information S2

## Data Availability

De‐identified data may be shared upon reasonable request to the corresponding author.
