# Supplementary material for: Corticosteroid Injection Practices of Australian Podiatrists for Common Foot and Ankle Conditions
Source: J Foot Ankle Res. 2026 Sep 11;19(3):e70214. doi: 10.1002/jfa2.70214 (PMC13570197; doi:10.1002/jfa2.70214)
Supplement: Supplementary file 1 [file JFA2-19-e70214-s001.pdf]

# Informed Consent

Current practice of corticosteroid injection for common foot and ankle conditions in Australia  The research is being carried out by the following researchers:

Role

Name

Organisation

Chief investigator

Glen Whittaker

La Trobe University, School of Allied Health Human Services and Sport

Co-investigator

Naomi Blood

Toorak Village Podiatry

Co-investigator

Matthew Cotchett

La Trobe University, School of Allied Health Human Services and Sport

Student investigator

Christopher Couesnon

La Trobe University, School of Allied Health Human Services and Sport

Research funder

This research is supported by in kind support by La Trobe University.

1. What is the study about?

You are invited to participate in a study of the current practice of corticosteroid injection for common foot and ankle conditions by Australian health professionals (such as foot and ankle surgeons, radiologists, rheumatologists, and podiatrists). We hope to learn about the corticosteroid, local anaesthetics, and injection techniques that are being used in Australia for common foot and ankle conditions.

2. Do I have to participate?

Being part of this study is voluntary. If you want to be part of the study we ask that you read the information below carefully.

You can read the information below and decide at the end if you do not want to participate. If you decide not to participate this won't affect your relationship with La Trobe University or any other listed organisation.

3. Who is being asked to participate?

You have been asked to participate because you:

- Are a health professional registered with AHPRA
- Are an Australian resident
- Use a primary language that is the same as the researchers
- Have administered at least 1 corticosteroid injection for a foot or ankle condition in the past 6 months.

4. What will I be asked to do?

If you want to take part in this study, we will ask you to complete an online survey answering questions about how you use corticosteroid injection in your clinical practice. It will take approximately 10-15 minutes of your time to be part of this study.

5. What are the benefits?

There is minimal benefit to you taking part in this research, however you have an opportunity to provide meaningful data about how health professionals use corticosteroid injection in Australia. The expected benefits to society in general are understanding how Australian health professionals use corticosteroid injection to manage common foot and ankle conditions, which can inform future randomsied trials comparing the effectiveness of corticosteroids and injection techniques.

6. What are the risks?

With any study there are (1) risks we know about, (2) risks we don't know about, and (3) risks we don't expect. If you experience something that you aren't sure about, please contact us immediately so we can discuss the best way to manage your concerns.

Name/Organisation

Position

Telephone

Email


Glen Whittaker

Senior Lecturer

+613 9479 5785

g.whittaker@latrobe.edu.au


We do not foresee any risks associated with this study.

7. What will happen to information about me?

By clicking on the 'I agree, start questionnaire' button, this tells us you want to take part in the study.

We will collect information about you in ways that will not reveal who you are.

We will store information about you in ways that will not reveal who you are.

We will publish information about you in ways that will not be identified in any type of publication from this study.

We will keep your information for an indefinite period after the project is completed because we may use your data for future closely related research. We will destroy all of your data if we deem it cannot be used for future research.

The storage, transfer and destruction of your data will be undertaken in accordance with the Research Data Management Policy https://policies.latrobe.edu.au/document/view.php?id=106/.

The personal information you provide will be handled in accordance with applicable privacy laws, any health information collected will be handled in accordance with the Health Records Act 2001 (Vic). Subject to any exceptions in relevant laws, you have the right to access and correct your personal information by contacting the research team.

8. Will I hear about the results of the study?

Yes you can hear about the results of the study through: (i) reading the publication associated with this research, or (ii) you can contact the Chief Investigator who can discuss the results of the study with you.

9. What if I change my mind?

If you no longer want to complete the questionnaire, simply close the web browser. If you change your mind after clicking on the 'Submit' button, we cannot withdraw your responses because we cannot link who you are with your questionnaire responses.

Your decision to withdraw at any point will not affect your relationship with La Trobe University or any other organisation listed.

10. Who can I contact for questions or want more information?

If you would like to speak to us, please use the contact details below:

Name/Organisation

Position

Telephone

Email

Glen Whittaker

Senior Lecturer

+613 9479 5785

g.whittaker@latrobe.edu.au

11. What if I have a complaint?

If you have a complaint about any part of this study, please contact:

Ethics Reference Number

Position

Telephone

Email

HEC24151

Senior Research Ethics Officer

+61 3 9479 1443

humanethics@latrobe.edu.au

You can download a copy of the Participant Information and Consent Form by clicking the attachment link below.

[Attachment: "PICF V4.docx"]

## Accept inclusion criteria

| | | Yes | No |
|---|---|---|---|
| 1) | Are you a health professional registered with AHPRA? | ☐ | ☐ |
| 2) | Are you an Australian resident? | ☐ | ☐ |
| 3) | Have you administered at least 1 corticosteroid injection for a foot and ankle condition in the past 6 months? | ☐ | ☐ |

4) I would like my information collected for this research to be:

◯ Only used for this specific study
◯ Used for future related studies

5) I (the participant) have read and understood the Participant Information Statement, and any questions have been answered to my satisfaction. I agree to participate in the study. I agree information provided by me or with my permission during the project may be included in a thesis, presentation and published in journals on the condition that I cannot be identified.

◯ Yes, I consent and I can start the survey
◯ No, I do not consent or wish to participate

# Demographic

What is your age

__________________________________

How do you currently describe your gender identity?

- ◯ Man/boy
- ◯ Woman/girl
- ◯ Non-binary
- ◯ I prefer to self describe
- ◯ I prefer not to answer

Please describe your gender

__________________________________

What is your health profession?

- ◯ Orthopaedic surgeon
- ◯ General Practitioner
- ◯ Sports Medicine Specialist
- ◯ Radiologist
- ◯ Rheumatologist
- ◯ Podiatrist (endorsed to prescribe scheduled medicines)
- ◯ Podiatric surgeon (endorsed to prescribe scheduled medicines)
- ◯ Nurse Practitioner

In which state or territory is your primary place of practice?

- ◯ Australian Capital Territory
- ◯ New South Wales ◯ Northern Territory
- ◯ Queensland ◯ South Australia
- ◯ Tasmania ◯ Victoria
- ◯ Western Australia

How many years have you worked as this health professional?

1 ———————————————— 80

*(Place a mark on the scale above)*

How many year's experience do you have administering corticosteroid injections?

0 ———————————————— 80

*(Place a mark on the scale above)*

How did you learn to administer corticosteroid injections? (select all that apply)

- ☐ Competency within an award course (e.g. Bachelor, Masters) required for registration
- ☐ Non-award courses (e.g. seminars, short courses) not required for registration
- ☐ Mentoring in a clinical environment
- ☐ Peer support in a clinical environment
- ☐ Self-taught
- ☐ Other

Please describe the other approach you used to learn how to administer corticosteroid injections.

__________________________________

**When deciding if a corticosteroid injection is appropriate, rank the following patient factors in order of their importance**

| | 1st | 2nd | 3rd | 4th | 5th | 6th | 7th | 8th | 9th | 10th |
|---|---|---|---|---|---|---|---|---|---|---|
| Pain severity (intensity + activity limitation) | ○ | ○ | ○ | ○ | ○ | ○ | ○ | ○ | ○ | ○ |
| Activity levels | ○ | ○ | ○ | ○ | ○ | ○ | ○ | ○ | ○ | ○ |
| Body mass index (BMI) | ○ | ○ | ○ | ○ | ○ | ○ | ○ | ○ | ○ | ○ |
| Immune status | ○ | ○ | ○ | ○ | ○ | ○ | ○ | ○ | ○ | ○ |
| Occupation | ○ | ○ | ○ | ○ | ○ | ○ | ○ | ○ | ○ | ○ |
| Medical history | ○ | ○ | ○ | ○ | ○ | ○ | ○ | ○ | ○ | ○ |
| Medication history | ○ | ○ | ○ | ○ | ○ | ○ | ○ | ○ | ○ | ○ |
| Whether evidence supports a corticosteroid injection for the condition | ○ | ○ | ○ | ○ | ○ | ○ | ○ | ○ | ○ | ○ |
| Patient preference | ○ | ○ | ○ | ○ | ○ | ○ | ○ | ○ | ○ | ○ |
| Outcome of past corticosteroid injection | ○ | ○ | ○ | ○ | ○ | ○ | ○ | ○ | ○ | ○ |

---

Do you consider other factors as important when deciding if a corticosteroid injection is appropriate?

○ Yes
○ No

---

Please describe the other patient factors

__________________________________

---

What is the maximum number of corticosteroid injections you would recommend a person receive per year?

__________________________________

---

What is the usual time interval you recommend waiting between corticosteroid injections? (Please enter a time interval in months).

__________________________________

---

Please describe how you normally prepare the injection site when administering a corticosteroid injection.

__________________________________________

---

When you administer a corticosteroid injection, do you usually have someone assisting you (e.g. sonographer, assistant etc)?

○ Yes
○ No

---

What is the total amount of money received by you/your service, per patient, when administering a corticosteroid injection? Please include the total amount, including patient out-of-pocket costs plus rebates received (e.g. Medicare).

If you work in a setting where there is no cost to the patient, please include any rebates received (e.g. Medicare), and if none are received for a corticosteroid injection, write 0.

__________________________________

# Cultural background

The following questions related to your cultural background.

| | |
|---|---|
| What is your ethnicity? | ◯ Oceanian<br>◯ North-West European<br>◯ Southern and Eastern European<br>◯ North African and Middle Eastern<br>◯ South-East Asian<br>◯ North-East Asian<br>◯ Southern and Central Asian<br>◯ Peoples of the Americas<br>◯ Sub-Saharan African |
| Oceanian | ◯ Australian Peoples<br>◯ New Zealand Peoples<br>◯ Melanesian and Papuan<br>◯ Micronesian<br>◯ Polynesian |
| North-West European | ◯ British<br>◯ Irish<br>◯ Western European<br>◯ Northern European |
| Southern and Eastern European | ◯ Southern European<br>◯ South Eastern European<br>◯ Eastern European |
| North African and Middle Eastern | ◯ Arab<br>◯ Jewish<br>◯ Peoples of the Sudan<br>◯ Other North African and Middle Eastern |
| South-East Asian | ◯ Mainland South-East Asian<br>◯ Maritime South-East Asian |
| North-East Asian | ◯ Chinese Asian<br>◯ Other North-East Asian |
| Southern and Central Asian | ◯ Southern Asian<br>◯ Central Asian |
| Peoples of the America's | ◯ North American<br>◯ South American<br>◯ Central American<br>◯ Caribbean Islander |
| Sub-Saharan African | ◯ Central and West African<br>◯ Southern and East African |
| Australian Peoples | ◯ Australian<br>◯ Australian Aboriginal<br>◯ Australian South Sea Islander<br>◯ Torres Strait Islander<br>◯ Norfolk Islander |
| New Zealand Peoples | ◯ Maori<br>◯ New Zealander |

| | |
|---|---|
| Melanesian and Papuan | ◯ New Caledonian<br>◯ Ni-Vanuatu<br>◯ Papa new Guinean<br>◯ Solomon Islander |
| Micronesian | ◯ I-Kiribati<br>◯ Nauruan |
| Polynesian | ◯ Cook Islander<br>◯ Fijian<br>◯ Niuean<br>◯ Samoan<br>◯ Tongan<br>◯ Hawaiian<br>◯ Tahitian<br>◯ Tokelauan<br>◯ Tuvaluan<br>◯ Pitcairn |
| British | ◯ English<br>◯ Scottish<br>◯ Welsh<br>◯ Channel Islander<br>◯ Manx<br>◯ British |
| Irish | ◯ Irish |
| Western European | ◯ Austrian<br>◯ Dutch<br>◯ Flemish<br>◯ French<br>◯ German<br>◯ Swiss<br>◯ Belgian<br>◯ Frisian<br>◯ Luxemburg |
| Northern European | ◯ Danish<br>◯ Finnish<br>◯ Icelandic<br>◯ Norwegian<br>◯ Swedish |
| Southern European | ◯ Basque<br>◯ Catalan<br>◯ Italian<br>◯ Maltese<br>◯ Portuguese<br>◯ Spanish<br>◯ Gibraltarian |

| | |
|---|---|
| South Eastern European | ◯ Albanian<br>◯ Bosnian<br>◯ Bulgarian<br>◯ Croatian<br>◯ Greek<br>◯ Macedonian<br>◯ Moldovan<br>◯ Montenegrin<br>◯ Romainian<br>◯ Roma Gypsy<br>◯ Serbian<br>◯ Slovene<br>◯ Cypriot<br>◯ Vlach |
| Eastern European | ◯ Belarusan<br>◯ Czech<br>◯ Estonian<br>◯ Hungarian<br>◯ Latvian<br>◯ Lithuanian<br>◯ Polish<br>◯ Russian<br>◯ Slovak<br>◯ Ukranian<br>◯ Sorb/Wend |
| Arab | ◯ Algerian<br>◯ Egyptian<br>◯ Iraqi<br>◯ Jordanian<br>◯ Kuwaiti<br>◯ Lebanese<br>◯ Libyan<br>◯ Moroccan<br>◯ Palestinian<br>◯ Saudi Arabian<br>◯ Syrian<br>◯ Tunisian<br>◯ Yemeni<br>◯ Bahraini<br>◯ Emirati<br>◯ Omani<br>◯ Qatari |
| Jewish | ◯ Jewish |
| Peoples of the Sudan | ◯ Bari<br>◯ Darfur<br>◯ Dinka<br>◯ Nuer<br>◯ South Sudanese<br>◯ Sudanese |

| | |
|---|---|
| Other North African and Middle Eastern | ◯ Berber<br>◯ Coptic<br>◯ Iranian<br>◯ Kurdish<br>◯ Turkish<br>◯ Assyrian<br>◯ Chaldean<br>◯ Mandaean<br>◯ Nubian<br>◯ Yezidi |
| Mainland South-East Asian | ◯ Anglo-Burmese<br>◯ Burmese<br>◯ Hmong<br>◯ Khmer (cambodian)<br>◯ Lao<br>◯ Thai<br>◯ Vietnamese<br>◯ Karen<br>◯ Mon<br>◯ Chin<br>◯ Rohingya |
| Maritime South-East Asian | ◯ Filipino<br>◯ Indoneasian<br>◯ Javanese<br>◯ Madurese<br>◯ Malay<br>◯ Sundanese<br>◯ Timorese<br>◯ Achnese<br>◯ Balinese<br>◯ Bruneian<br>◯ Kadazan<br>◯ Singaporean<br>◯ Temoq |
| Chinese Asian | ◯ Chinese<br>◯ Taiwanese |
| Other North-East Asian | ◯ Japanese<br>◯ Koran<br>◯ Mongolian<br>◯ Tibetan |

| | |
|---|---|
| Southern Asian | ◯ Anglo-Indian<br>◯ Bengali<br>◯ Burgher<br>◯ Gujarati<br>◯ Indian<br>◯ Malayali<br>◯ Nepalese<br>◯ Pakistani<br>◯ Punjabi<br>◯ Sikh<br>◯ Sinhalese<br>◯ Maldivian<br>◯ Bangladeshi<br>◯ Bhutanese<br>◯ Fijian Indian<br>◯ Kashmiri<br>◯ Parsi<br>◯ Sindhi<br>◯ Sri Lankin<br>◯ Sri lankin Tamil<br>◯ Indian Tamil<br>◯ Tamil<br>◯ Tlugu |
| Central Asian | ◯ Afghan<br>◯ Armenian<br>◯ Gerogian<br>◯ Kazakh<br>◯ Pathan<br>◯ Uzbek<br>◯ Azeri<br>◯ Hazara<br>◯ Tajik<br>◯ Tatar<br>◯ Turkmen<br>◯ Uighur<br>◯ Kyrgyz |
| North American | ◯ African American<br>◯ American<br>◯ Canadian<br>◯ French Canadian<br>◯ Hispanic North American<br>◯ Native North American<br>◯ Bermudan |
| South American | ◯ Argentinian<br>◯ Bolivian<br>◯ Brazilian<br>◯ Chilean<br>◯ Colombian<br>◯ Ecuadorian<br>◯ Guyanese<br>◯ Peruvian<br>◯ Uruguayan<br>◯ Venezuelan<br>◯ Paraguayan |

| | |
|---|---|
| Central American | ◯ Mexican<br>◯ Nicaraguan<br>◯ Salvadoran<br>◯ Costa Rican<br>◯ Guatemalan<br>◯ Mayan |
| Caribbean islander | ◯ Cuban<br>◯ Jamaican<br>◯ Trinidadian Tobagonian<br>◯ Barbadian<br>◯ Puerto Rican |
| Central and West African | ◯ Akan<br>◯ Fulani<br>◯ Ghanian<br>◯ Nigerian<br>◯ Yoruba<br>◯ Ivorean<br>◯ Liberian<br>◯ Sierra Leonean<br>◯ Acholi<br>◯ Cameroonian<br>◯ Congolese<br>◯ Gio<br>◯ Igbo<br>◯ Krahn<br>◯ Mandinka<br>◯ Senegalese<br>◯ Themne<br>◯ Togolese |

Southern and East African

- ◯ Afrikaner
- ◯ Angolan
- ◯ Eritrean
- ◯ Ethiopian
- ◯ Kenyan
- ◯ Malawian
- ◯ Mauritian
- ◯ Mozambican
- ◯ Namibian
- ◯ Oromo
- ◯ Seychellois
- ◯ Somali
- ◯ South African
- ◯ Tanzanian
- ◯ Ugandan
- ◯ Zambian
- ◯ Zimbabwean
- ◯ Amhara
- ◯ Batswana
- ◯ Hutu
- ◯ Masia
- ◯ Tigrayan
- ◯ Tigre
- ◯ Zulu
- ◯ Burundian
- ◯ Hunama
- ◯ Madi
- ◯ Ogaden
- ◯ Rwandan
- ◯ Shona
- ◯ Swahili
- ◯ Swazilander

# Plantar fascia injection

The following questions are about how you administer corticosteroid injection for plantar fascia conditions.

Do you use corticosteroid injection to manage plantar fascia conditions?

- ◯ Yes
- ◯ No

Please describe your reasons for not using corticosteroid injection for this condition.

__________________________________

On average, how many corticosteroid injections would you administer for plantar fascia conditions per month?

- ◯ 0-5
- ◯ 6-10
- ◯ 11-15
- ◯ 15+

What corticosteroid do you usually select as your first choice for a plantar fascia injection?

- ◯ Betamethasone 5.7 mg/mL (Celestone Chronodose)
- ◯ Methylprednisolone acetate 40 mg/mL (Depo-Medrol, Depo-Nisolone)
- ◯ Triamcinolone acetonide 10 mg/mL (Kenacort-A 10)
- ◯ Triamcinolone acetonide 40 mg/mL (Kenacort-A 40)
- ◯ Dexamethasone phosphate 4 mg/mL (Dexamethasone)
- ◯ Other

What is the other corticosteroid you usually use?

__________________________________

Please describe your clinical reasoning for selecting this corticosteroid.

__________________________________

What volume of corticosteroid do you usually inject? (If your choice is between responses (eg 0.7 mL), enter the higher volume (eg 1 mL).

- ◯ 0.5 mL
- ◯ 1 mL
- ◯ 1.5 mL
- ◯ 2 mL
- ◯ 2.5 mL or greater

Do you usually combine the corticosteroid with a local anaesthetic?

- ◯ Yes
- ◯ No

What local anaesthetic do you usually select for a plantar fascia injection?

- ◯ Lidocaine 1%
- ◯ Lidocaine 2%
- ◯ Bupivacaine (Marcain)
- ◯ Ropivacaine (Naropin)
- ◯ Other

Please describe the other local anaesthetic

__________________________________

Please describe your clinical reasoning for selecting this local anaesthetic.

__________________________________

What volume of anaesthetic do you usually inject? (If your choice is between responses (eg 0.7 mL), enter the higher volume (eg 1 mL).

- ◯ 0.5 mL
- ◯ 1 mL
- ◯ 1.5 mL
- ◯ 2 mL
- ◯ 2.5 mL or greater

| | |
|---|---|
| For a plantar fascia injection, from which site do you usually inject? | ◯ Medial heel<br>◯ Medial oblique heel<br>◯ Lateral heel<br>◯ Plantar heel<br>◯ Other |
| Please describe the other site you usually use to inject the plantar fascia | __________________________ |
| Do you usually perform a regional nerve block (e.g. tibial block) prior to your heel injection? | ◯ Yes<br>◯ No |
| Do you usually use a topical anaesthetic when administering a plantar fascia injection? | ◯ Yes<br>◯ No |
| Do you usually incorporate diagnostic imaging when planning your injection? | ◯ Yes<br>◯ No |
| What type of imaging do you usually use? | ◯ Ultrasound<br>◯ X - ray<br>◯ MRI<br>◯ Fluoroscopy<br>◯ Other |
| Please describe the other type of imaging | __________________________ |
| Do you usually use ultrasound guidance when administering a plantar fascia injection? | ◯ Yes<br>◯ No |
| Please describe any specific injection techniques you usually use (e.g. peppering) for plantar fascia corticosteroid injection. | ________________________________ |
| In your experience, on average, how long do patients usually experience a meaningful pain reduction? (Please provide your answer in months). | ◯ 0<br>◯ 1<br>◯ 2<br>◯ 3<br>◯ 6<br>◯ 12<br>◯ 18<br>◯ 24 |
| In your experience, how frequently do patients experience adverse effects following a plantar fascia injection? (Please respond as a percentage). | 0% 50% 100%<br>*(Place a mark on the scale above)* |
| In your experience, what is the most frequent adverse effect for a plantar fascia injection? | __________________________ |

# Intermetatarsal injection

The following questions are about how you administer corticosteroid injection for intermetatarsal conditions (e.g. neuroma, bursa).

---

Do you use corticosteroid injection to manage intermatatarsal conditions (e.g. neuroma, bursa)?

- ◯ Yes
- ◯ No

---

Please describe your reasons for not using corticosteroid injection for this condition.

__________________________________

---

On average, how many corticosteroid injections would you administer for intermetatarsal conditions per month?

- ◯ 0-5
- ◯ 6-10
- ◯ 11-15
- ◯ 15+

---

What corticosteroid do you usually select as your first choice for an intermatatarsal injection?

- ◯ Betamethasone 5.7 mg/mL (Celestone Chronodose)
- ◯ Methylprednisolone acetate 40 mg/mL (Depo-Medrol, Depo-Nisolone)
- ◯ Triamcinolone acetonide 10 mg/mL (Kenacort-A 10)
- ◯ Triamcinolone acetonide 40 mg/mL (Kenacort-A 40)
- ◯ Dexamethasone phosphate 4 mg/mL (Dexamethasone)
- ◯ Other

---

What is the other corticosteroid you usually use?

__________________________________

---

Please describe your clinical reasoning for selecting this corticosteroid.

__________________________________

---

What volume of corticosteroid do you usually inject? (If your choice is between responses (eg 0.7 mL), enter the higher volume (eg 1 mL).

- ◯ 0.5 mL
- ◯ 1 mL
- ◯ 1.5 mL
- ◯ 2 mL
- ◯ 2.5 mL or greater

---

Do you usually combine the corticosteroid with a local anaesthetic?

- ◯ Yes
- ◯ No

---

What local anaesthetic do you usually select for an intermetatarsal injection?

- ◯ Lidocaine 1%
- ◯ Lidocaine 2%
- ◯ Bupivacaine (Marcain)
- ◯ Ropivacaine (Naropin)
- ◯ Other

---

Please describe the other local anaesthetic

__________________________________

---

Please describe your clinical reasoning for selecting this local anaesthetic.

__________________________________

---

What volume of anaesthetic do you usually inject? (If your choice is between responses (eg 0.7 mL), enter the higher volume (eg 1 mL).

- ◯ 0.5 mL
- ◯ 1 mL
- ◯ 1.5 mL
- ◯ 2 mL
- ◯ 2.5 mL or greater

| | |
|---|---|
| For an intermetatarsal injection, from which site do you usually inject? | ◯ Dorsal approach (perpendicular to the toes)<br>◯ Plantar approach (perpendicular to the toes)<br>◯ Webspace (parallel with the toes)<br>◯ Other |
| Please describe the other site you usually use to inject | __________________________________ |
| Do you usually perform a regional nerve block (e.g. tibial block) prior to your injection? | ◯ Yes<br>◯ No |
| Do you usually use a topical anaesthetic when administering an intermetatarsal injection? | ◯ Yes<br>◯ No |
| Do you usually incorporate diagnostic imaging when planning your injection? | ◯ Yes<br>◯ No |
| What type of imaging do you usually use? | ◯ Ultrasound<br>◯ X - ray<br>◯ MRI<br>◯ Fluoroscopy<br>◯ Other |
| Please describe the other type of imaging | __________________________________ |
| Do you usually use ultrasound guidance when administering an intermetatarsal injection? | ◯ Yes<br>◯ No |
| Please describe any specific injection techniques you usually use (e.g. peppering) for intermetatarsal corticosteroid injection. | __________________________________________ |
| In your experience, on average, how long do patients usually experience a meaningful pain reduction? (Please provide your answer in months). | ◯ 0<br>◯ 1<br>◯ 2<br>◯ 3<br>◯ 6<br>◯ 12<br>◯ 24 |
| In your experience, how frequently do patients experience adverse effects following an intermetatarsal injection? (Please respond as a percentage). | 0% 50% 100%<br>(Place a mark on the scale above) |
| In your experience, what is the most frequent adverse effect for an intermetatarsal injection? | __________________________________ |

# Intra-articular injection

The following questions are about how you administer corticosteroid injection for Intra-articular conditions (e.g. arthritis).

| | |
|---|---|
| Do you use corticosteroid injection to manage intra-articular conditions (e.g. arthritis)? | ◯ Yes<br>◯ No |
| Please describe your reasons for not using corticosteroid injection for intra-articular conditions. | __________________________________ |
| On average, how many corticosteroid injections would you administer for intra-articular conditions per month? | ◯ 0-5<br>◯ 6-10<br>◯ 11-15<br>◯ 15+ |
| What corticosteroid do you usually select as your first choice for an intra-articular injection? | ◯ Betamethasone 5.7 mg/mL (Celestone Chronodose)<br>◯ Methylprednisolone acetate 40 mg/mL (Depo-Medrol, Depo-Nisolone)<br>◯ Triamcinolone acetonide 10 mg/mL (Kenacort-A 10)<br>◯ Triamcinolone acetonide 40 mg/mL (Kenacort-A 40)<br>◯ Dexamethasone phosphate 4 mg/mL (Dexamethasone)<br>◯ Other |
| What is the other corticosteroid you usually use? | __________________________________ |
| Please describe your clinical reasoning for selecting this corticosteroid. | __________________________________ |
| Do you usually select different corticosteroids depending on the foot/ankle joint you are injecting? | ◯ Yes<br>◯ No |
| Please describe your clinical reasoning for the selecting a corticosteroid for different foot/ankle joints. | __________________________________ |
| What volume of corticosteroid do you usually inject? (If your choice is between responses (eg 0.7 mL), enter the higher volume (eg 1 mL). | ◯ 0.5 mL<br>◯ 1 mL<br>◯ 1.5 mL<br>◯ 2 mL<br>◯ 2.5 mL or greater |
| Do you usually combine the corticosteroid with a local anaesthetic? | ◯ Yes<br>◯ No |
| What local anaesthetic do you usually select for an intra-articular injection? | ◯ Lidocaine 1%<br>◯ Lidocaine 2%<br>◯ Bupivacaine (Marcain)<br>◯ Ropivacaine (Naropin)<br>◯ Other |
| Please describe the other local anaesthetic | __________________________________ |
| Please describe your clinical reasoning for selecting this local anaesthetic. | __________________________________ |

| | |
|---|---|
| What volume of anaesthetic do you usually inject? (If your choice is between responses (e.g. 0.7 mL), enter the higher volume (e.g. 1 mL). | ◯ 0.5 mL<br>◯ 1 mL<br>◯ 1.5 mL<br>◯ 2 mL<br>◯ 2.5 mL or greater |
| Do you usually perform a regional nerve block (e.g. tibial block) prior to your injection? | ◯ Yes<br>◯ No |
| Do you usually use a topical anaesthetic when administering an intra-articular injection? | ◯ Yes<br>◯ No |
| Do you usually incorporate diagnostic imaging when planning your injection? | ◯ Yes<br>◯ No |
| What type of imaging do you usually use? | ◯ Ultrasound<br>◯ X - ray<br>◯ MRI<br>◯ Fluoroscopy<br>◯ Other |
| Please describe the other type of imaging | __________________________________ |
| Do you usually use ultrasound guidance when administering an intra-articular injection? | ◯ Yes<br>◯ No |
| Please describe any specific injection techniques you usually use (e.g. peppering) for intra-articular corticosteroid injection. | __________________________________________ |
| In your experience, on average, how long do patients usually experience a meaningful pain reduction? (Please provide your answer in months). | ◯ 0<br>◯ 1<br>◯ 2<br>◯ 3<br>◯ 6<br>◯ 12<br>◯ 24 |
| In your experience, how frequently do patients experience adverse effects following an intra-articular injection? (Please respond as a percentage). | 0% 50% 100%<br>(Place a mark on the scale above) |
| In your experience, what is the most frequent adverse effect for an intermetatarsal injection? | __________________________________ |
